# Influence of high ovarian hormones on QT interval duration in young African women

**DOI:** 10.1002/phy2.263

**Published:** 2014-03-20

**Authors:** Edwige Balayssac‐Siransy, Soualiho Ouattara, Anicet Adoubi, Chantal Kouamé, Marie‐Laure Hauhouot‐Attoungbré, Cyrille Dah, Pascal Bogui

**Affiliations:** ^1^ Service des Explorations Fonctionnelles Centre hospitalier universitaire de Yopougon 21 BP 632 Abidjan Côte d'ivoire; ^2^ Laboratoire de Physiologie et d'Explorations Fonctionnelles Unité de Formation et de Recherche en Sciences Médicales Université Félix Houphouët Boigny 01 BPV 34 Abidjan Côte d'ivoire; ^3^ Service de Cardiologie du Centre Hospitalier Universitaire de Bouaké 02 BP 801 Abidjan Côte d'ivoire; ^4^ Laboratoire d'analyses biologiques de l'Institut de Cardiologie d'Abidjan BP 206 Abidjan Côte d'Ivoire; ^5^ Service des Explorations Fonctionnelles Centre hospitalier universitaire de Cocody BPV 13 Abidjan Côte d'Ivoire

**Keywords:** African, cardiac repolarization, menstrual cycle

## Abstract

The longer QT interval duration observed in women compared to men is usually attributed to sexual hormones. The aim of our study was to investigate, among black African women, the influence of hormonal variations during the menstrual cycle on the duration of the QT interval. Fourteen young black African women, healthy, sedentary, aged 24 ± 1.7 years, with a regular menstrual cycle (28 ± 1 days) were selected from 59 volunteers. At each phase of their menstrual cycle, menstrual 2.9 ± 0.6 days, follicular 13 ± 1.5 days, and luteal 23.1 ± 1.4 days, an electrocardiogram was performed in supine position after a resting period of 30 min, to measure QT interval duration. QT interval was corrected by Bazett's (QTc_b_) and Fridericia's (QTc_f_) formulae. Then, blood samples were obtained to measure estradiol, progesterone, and serum electrolytes (K^+^, Ca^2+^, Mg^2+^). There was no significant difference in uncorrected QT intervals between the three phases of the menstrual cycle. It was the same for QTc_b_ and QTc_f_. Moreover, during the menstrual cycle, we did not observe any correlation between each QT, QTc_b_, QTc_f_, and estradiol levels which raised during the follicular phase (356.61 ± 160.77 pg/mL) and progesterone levels which raised during the luteal phase (16.38 ± 5.88 ng/mL). Finally, the method of Bland and Altman demonstrated that the corrections of QT by Bazett and Fridericia formulae were not interchangeable. The results of this study showed that high levels of estradiol and progesterone in young black African women did not influence the QT, QTc_b_ and QTc_f_ intervals duration during the menstrual cycle.

## Introduction

QT interval measured at rest by electrocardiography represents the duration of ventricular repolarization. Since the work of Bazett (1920), several studies have reported a longer QT interval in women than in men (Adams 1936; Goldberg et al. 1991; Rautaharju et al. 1992; Makkar et al. 1993; Molnar et al. 1996; Lehmann et al. 1996; Burke et al. 1997; Stramba‐Badiale et al. 1997; Drici et al. 1998; Surawicz and Parikh 2002). This gender difference was not found between boys and girls before puberty (Sutliff and Holt 1925; Iliff and Lee 1952; Rijnbeek et al. 2001). These observations led some authors to investigate the role of sexual hormones on QT interval duration during the menstrual cycle (Burke et al. 1997; Hulot et al. 2003; Endres et al. 2004; Nakagawa et al. 2005). These studies were also motivated by the discovery of the inhibitory effect of estradiol on potassium channels that regulate the repolarization phase of the action potential, leading to QT interval prolongation in animals (Drici et al. 1996; Ebert et al.^1998^). However, no change in QT interval has been reported with hormonal fluctuations during the menstrual cycle in Caucasian women (Burke et al. 1997; Hulot et al. 2003; Nakagawa et al.^2006^). These authors implicated the low levels of estradiol in Caucasian women who participated in their study compared to the high estradiol concentration in Drici's study which increased QT interval in animals (Drici et al. 1996). This suggested that a high level of estradiol in women could lead to a long QT interval. Marsh et al. (2011), after measuring estradiol and progesterone each day during one menstrual cycle, reported significantly greater level of estradiol in the late follicular phase in African‐American women than in Caucasian women but no significant difference in progesterone level between the two groups. This great estradiol level in the late follicular phase of these women could lead to a long QT interval.

Also, the purpose of this study was to investigate in young black African women, the influence of hormonal variations during the menstrual cycle on QT interval duration.

## Materials and Methods

### Ethical approval

The study was conducted in accordance with the guidelines set by the Declaration of Helsinki and was approved by the Ethics Committee of the Academic Hospital of Yopougon (Abidjan, Ivory Coast).

All patients were advised about the purpose and procedures of the study, and they gave their informed written consent.

### Population

Among 59 urban young black African women volunteers, 21 were selected (20–30 years) and had a regular menstrual cycle for more than 3 months (28 ± 1 days). They were sedentary, nonsmokers and showed no cardiovascular or respiratory disease. They have never been pregnant and did not take oral contraceptives or medications that could affect cardiac repolarization. They had a clinical examination, normal resting electrocardiogram with a CardioPlug (CARDIONICS S.A., Brussels, Belgium), and a normal ionic balance. No family history of sudden death has been found in this population.

### Experimental protocol

Each woman was admitted in the function tests unit of the Academic Hospital of Yopougon, at each phase of their menstrual cycle: menstrual (days 1–5), follicular (4 days preceding ovulation), and luteal (days 6–10 after ovulation). These phases were identified taking into account the onset of menses and the date of ovulation (calculated from the known length of the menstrual cycle and fixed at 14 days for luteal phase). Subjects were explored in the morning between 8:00 a.m. and 12:00 a.m. Each woman, topless, was installed on an examination bed, in an air‐conditioned room at 22°C. A clinical examination was performed to confirm the absence of cardiorespiratory event. Then, still in the supine position, blood pressure was measured on the left arm with an electronic monitor (OMRON M6, Kyoto, Japan) and an electrocardiogram was performed with a CardioPlug (CARDIONICS S.A., Brussels, Belgium) as recommended by the AHA/ACCF/HRS 2007 (Kligfield et al. 2007). The electrocardiograph was programmed to 25 mm/s, 10 mV/mm, with a long DII derivation. Subjects were covered from neck to feet and remained quietly in supine position, eyes closed for 30 consecutive minutes before starting the recordings of the electrocardiogram (ECG), the blood pressure and the heart rate. Blood samples were collected in a dry tube for the determination of ovarian hormones (estradiol and progesterone) and in a heparin tube for the measurements of electrolytes (K^+^, Ca^2+^, Mg^2+^). Estradiol and progesterone levels were determined by immunological method (mini VIDAS, Biomerieux, Paris, France). At each of the three phases of the menstrual cycle, all explorations were made at the same time, in the same room, and under the same experimental conditions.

All electrocardiographic recordings were read by the same investigator who was blinded to the phases of the menstrual cycle and to the results of hormones levels. The QT intervals were measure manually in long DII derivation.

### Parameters

All QT intervals were measured on each electrocardiogram. The QT interval was measured from the onset of the QRS complex to the end of the T wave. The end of the T wave was obtained by extending a tangent from the T wave immediately before the P or U and extending it to the level of the isoelectric baseline (Kadish et al. 1990). Each RR interval preceding each QT interval was measured in millimeters and converted to seconds. Each QT interval measured was corrected (QTc) using Bazett (1920) [QTc_b_ (ms) = QT/RR^1/2^] and Fridericia (1920) [QTc_f_ (ms) = QT/RR^1/3^] formulae. In each subject, we calculated the difference between QT interval in follicular phase and QT interval in luteal phase. The same procedure was done for QTc_b_ and QTc_f_.

### Statistical tests

The minimum sample size needed (>12 subjects) was calculated to allow a detection of a mean difference of 16 ms in the duration of QT interval between two of the three phases of the menstrual cycle with *α* = 0.05 and power = 0.80. The SD of this difference was estimated from prior data to be 15–22 ms (Drici et al. 1996; Hulot et al. 2003). Data were expressed as mean ± standard deviation. A one‐way analysis of variance (ANOVA) for repeated measures was used to compare the different parameters measured between the three phases of the menstrual cycle. The correlations of QT, QTc_b_, and QTc_f_ intervals with hormones and serum electrolytes levels were tested using the Pearson correlation coefficient. Interchangeability between the QT corrected by Bazett and QT corrected by Fridericia was investigated by the method of Bland and Altman (1986). To test whether the mean difference between QTc_b_ and QTc_f_ was not significantly different from 0, a one‐sample Student *t*‐test procedure was performed. Finally, a linear regression was drawn on the Bland‐Altman scatter plot to assess possible changes in bias with the increase in QTc interval. The acceptable limit of agreement to assess the interchangeability between the two corrections of QT interval was fixed at 15 ms of the mean values. Above this limit, the physiological meanings of the measurement could be considered doubtful. Statistical significance was determined by *P*‐value <0.05. Analyses were conducted using SPSS (version 17, Chicago, IL).

## Results

### Population characteristics

Among the 21 young black African women selected for this study, hormonal assays confirmed the three phases of the menstrual cycle in 14 of them. These assays were characterized by low levels of estradiol and progesterone in menstrual phase, an estradiol peak and low progesterone level in the follicular phase and, in the luteal phase, a progesterone peak above 5 ng/mL, and an oestradiol level under the follicular peak. These 14 young women selected for the study had a mean age of 24 ± 1.7 years and an average menstrual cycle period of 28 ± 1 days. They were explored during menstrual phase 2.9 ± 0.6 days, follicular phase 13 ± 1.5 days, and luteal phase 23.1 ± 1.4 days. The characteristics of the 14 young women during the three phases of the menstrual cycle are reported in Table 1. Resting heart rate was significantly higher in the luteal phase. Resting diastolic blood pressure was significantly lower in the luteal phase. There was no statistical difference for systolic blood pressure and serum electrolytes (K^+^, Ca^2+^, Mg^2+^) between menstrual, follicular, and luteal phases.

**Table 1:** Population characteristics

Parameters	Menstrual phase *n* = 14	Follicular phase *n* = 14	Luteal phase *n* = 14	*P*
BMI, kg/m²	21.9 ± 3.5	21.6 ± 3.3	21.6 ± 3.4	0.18
HR, beats/min	69 ± 15	68 ± 14	74 ± 13	0.01
SBP, mmHg	103 ± 6	103 ± 9	102 ± 7	0.47
DBP, mmHg	71 ± 5	70 ± 6	68 ± 6	0.03
K^+^, mEq/L	4.3 ± 0.2	4.5 ± 0.3	4.4 ± 0.3	0.41
Ca^2+^, mg/L	96 ± 5	96 ± 4	95 ± 3	0.94
Mg^2+^, g/L	18 ± 1	18 ± 1	18 ± 1	0.24

Data are presented as mean ± SD.

BMI, body mass index; HR, heart rate; SBP, systolic blood pressure; DBP, diastolic blood pressure; *P*,* P*‐value.

### QT, QTc_b_, and QTc_f_ intervals duration

The average lengths of QT, QTc_b_, and QTc_f_ in the three phases of the menstrual cycle are reported in Table 2. There was no significant difference between the QT, QTc_b_, QTc_f_ intervals of the three phases of the menstrual cycle (*P* = 0.09; *P* = 0.98; *P* = 0.53, respectively). For each phase of the menstrual cycle, the average QTc_b_ value was significantly greater than the average value of QTc_f_ (menstrual *P* = 0.007; follicular *P* = 0.03; luteal *P* = 0.002).

**Table 2:** Average length of uncorrected and corrected QT intervals

Parameters	Menstrual phase *n* = 14	Follicular phase *n* = 14	Luteal phase *n* = 14	*P*
QT, ms	350 ± 20.6	354.6 ± 22.5	341.9 ± 18.7	0.09
QTc_b_, ms	381 ± 26.7	378.7 ± 26.3	379.3 ± 26.8	0.98
QTc_f_, ms	370 ± 17.5	370 ± 16.3	365.9 ± 16.7	0.53
*P* (QTc_b_/QTc_f_)	0.007	0.03	0.002	

Data are presented as mean ± SD.

QTc_b_, QT interval corrected by Bazett's formula; QTc_f_, QT interval corrected by Fridericia's formula; *P*,* P*‐value.

The mean difference in QT between luteal and follicular phases was −12.7 ± 14.9 ms. The mean differences in each QTc_b_ and QTc_f_ between luteal and follicular phases were 0.5 ± 10.7 ms and −4.1 ± 9.7 ms, respectively.

### RR intervals during the three phases of menstrual cycle

RR interval was significantly longer in the follicular phase than in the menstrual and luteal phases (899 ± 194; 863 ± 176; 826 ± 162 ms; *P* = 0.04).

### Hormone levels

The estradiol level was significantly higher in the follicular phase than in the menstrual and luteal phases (Table 3). The progesterone level was significantly greater in the luteal phase than in menstrual and follicular phases (Table 3).

**Table 3:** Serum estradiol and progesterone levels during the menstrual cycle

Parameters	Menstrual phase *n* = 14	Follicular phase *n* = 14	Luteal phase *n* = 14	*P*
Estradiol, pg/mL	49.57 ± 22.79	356.61 ± 160.77	245.18 ± 98.73	<10^−6^
Progesterone, ng/mL	0.56 ± 0.46	0.82 ± 0.49	16.38 ± 5.88	<10^−6^

Data are presented as mean ± SD.

*P*,* P*‐value.

### Relationship

#### QT, QTc_b_, QTc_f_ with RR intervals

**Figure 1:**
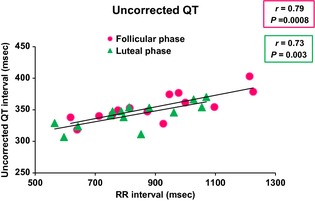
Uncorrected QT versus RR relationships during follicular and luteal phases of menstrual cycle (*n* = 14). During follicular and luteal phases of the menstrual cycle, there was a significant positive correlation between QT and RR intervals (*P* = 0.0008 and *P* = 0.003, respectively).

**Figure 2:**
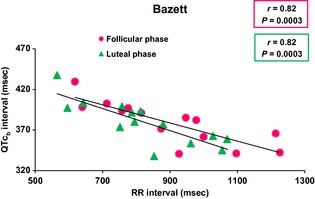
QT interval corrected by Bazett's formula (QTc_b_) versus RR relationship during follicular and luteal phases of menstrual cycle (*n* = 14). With Bazett's formula, QTc_b_ intervals showed a significant negative correlation with the RR intervals during the follicular and luteal phases (*P* = 0.0003 and *P* = 0.0003, respectively).

**Figure 3:**
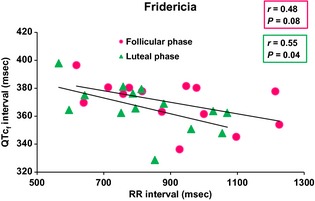
QT interval corrected by Fridericia's formula (QTc_f_) versus RR relationship during follicular and luteal phases of menstrual cycle (*n* = 14). With Fridericia's formula, there was a correlation between QTc_f_ intervals and RR intervals in luteal phase only (*P* = 0.04) but not in follicular phase (*P* = 0.08).

Figures 1, 2 and 3 showed the relationship of QT, QTc_b_, and QTc_f_ intervals_,_ respectively, with RR intervals, in the follicular and luteal phases of the menstrual cycle. There was a significant positive correlation between QT and RR intervals during the follicular (*r* = 0.79; *P* = 0.0008) and luteal (*r* = 0.73; *P* = 0.003) phases of the menstrual cycle. With Bazett's formula, QTc_b_ intervals showed a significant negative correlation with the RR intervals during the follicular (*r* = 0.82; *P* = 0.0003) and luteal (*r* = 0.82; *P* = 0.0003) phases. However, with Fridericia's formula, there was a significant correlation between QTc_f_ intervals and RR intervals in the luteal phase only (*r* = 0.55; *P* = 0.04) but not in the follicular phase (*r* = 0.48; *P* = 0.08).

#### QT, QTc_b_, and QTc_f_ intervals with serum electrolytes

There was no correlation between QT interval and serum potassium (*r* = 0.16; *P* = 0.58), calcium (*r* = 0.08; *P* = 0.78), and magnesium (*r* = 0.14; *P* = 0.63). It was the same for QTc_b_ (*r* = 0.12, *P* = 0.68; *r* = 0.3, *P* = 0.29; *r* = 0.19, *P* = 0.51) and QTc_f_ (*r* = 0.17, *P* = 0.5; *r* = 0.26, *P* = 0.36; *r* = 0.14, *P* = 0.63).

#### QT, QTc_b_,QTc_f_ intervals with hormone levels

**Figure 4:**
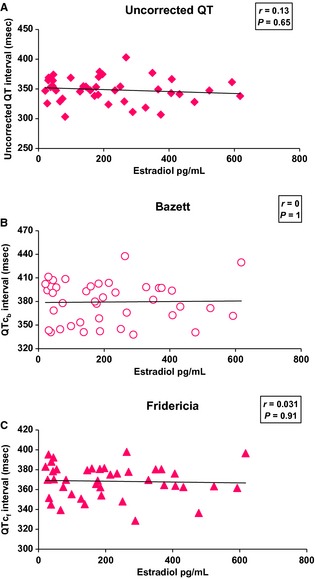
Correlation between uncorrected QT (A), QTc_b_ (B), QTc_f_ (C) interval and estradiol during the menstrual cycle (*n* = 14). No correlation was found between the QT, QTc_b_, QTc_f_ intervals and estradiol (*P* = 0.65; *P* = 1; *P* = 0.91, respectively).

**Figure 5:**
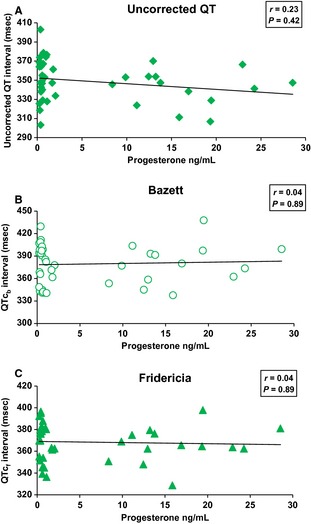
Correlation between uncorrected QT (A), QTc_b_ (B), QTc_f_ (C) intervals and progesterone during the menstrual cycle (*n* = 14). No correlation was found between the QT, QTc_b_, QTcf intervals and progesterone (*P* = 0.42; *P* = 0.89; *P* = 0.89, respectively).

No correlation was found between the QT, QTc_b_, QTc_f_ intervals and estradiol during the menstrual cycle (*r* = 0.13, *P* = 0.65; *r* = 0, *P* = 1; *r* = 0.03, *P* = 0.91) (Fig. 4 A, B, and C). It was the same with progesterone (*r* = 0.23, *P* = 0.42; *r* = 0.04, *P* = 0.89; *r* = 0.04, *P* = 0.89) (Fig. 5 A, B, and C).

#### QTc_b_ and QTc_f_ and comparison of QTc_b_ and QTc_f_ by the method of Bland‐Altman

**Figure 6:**
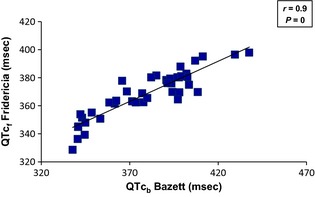
Correlation between uncorrected QTc_b_ (Bazett) and QTc_f_ (Fridericia) during the three phases of menstrual cycle (*n* = 14). During the menstrual cycle, there was a significant linear correlation between QTc_b_ intervals corrected by the Bazett's formula and QTc_f_ intervals corrected by the Fridericica's formula (*P* = 0).

During the menstrual cycle, there was a significant correlation between QTc_b_ and QTc_f_ intervals (*r* = 0.9; *P* = 0) (Fig. 6).

**Figure 7:**
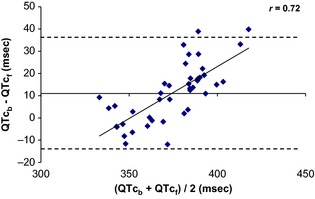
Bland and Altman analysis on QT corrected by Bazett's formula (QTc_b_) and Fridericia's formula (QTc_f_) (*n* = 14). There was no interchangeability between formulae of Bazett and Fridericia and a trend for an increase in the differences between the two corrections of prediction with the increase of QTc intervals.

The results of the Bland and Altman analysis are displayed in Fig. 7. The bias (11.4 ms) was significantly different from 0 (*P* < 0.05) suggesting a mean difference between QTc_b_ and QTc_f_. The significant positive linear regression showed a tendency to increase bias values with corrected QT values (*r* = 0.72; *P* = 0.003). The upper limit of agreement after correction by the bias (36.92–11.04 = 25.88 ms) was above the approval limit proposed 15 ms. In conclusion, there was no interchangeability between Bazett and Fridericia formulae.

## Discussion

This study investigated the influence of ovarian hormones on the QT interval duration during the menstrual cycle in young black African woman. Despite significantly higher estradiol level in the follicular phase and greater progesterone level in the luteal phase, we did not observe any significant difference in the duration of each QT, QTc_b_ (Bazett), and QTc_f_ (Fridericia) between the three phases of the menstrual cycle. Moreover, there was no correlation between QT, QTc_b_, QTc_f_ intervals and the level of ovarian hormones (estradiol and progesterone). Our results suggest no influence of ovarian hormones on the duration of the QT, QTc_b_, and QTc_f_ intervals during the menstrual cycle in young black African woman.

The role of ovarian hormones on QT interval duration has been previously investigated due to a longer QT interval observed in women than in men, from puberty to menopause (Adams 1936; Goldberg et al. 1991; Rautaharju et al. 1992; Makkar et al. 1993; Molnar et al. 1996; Lehmann et al. 1996; Burke et al. 1997; Stramba‐Badiale et al. 1997; Drici et al. 1998; Surawicz and Parikh 2002). In agreement with our results, several studies (Burke et al. 1997; Rodriguez et al. 2001; Hulot et al. 2003 et Endres et al. 2004) reported similar QT interval duration in the different phases of the menstrual cycle, in 17–22 Caucasian women. The peak estradiol levels found by these authors during the follicular phase were: 122 ± 42 pg/mL (Rodriguez et al. 2001), 204.3 ± 75.45 pg/mL (Hulot et al. 2003) and 70 ± 20 pg/mL (Nakagawa et al. 2006) lower than ours (356.61 ± 160.77 pg/mL). In the luteal phase, our estradiol peak (245.18 ± 98.73 pg/mL) was higher than that in their study with 123 ± 49 pg/mL (Rodriguez et al. 2001) or 78 ± 11 pg/mL (Nakagawa et al. 2006). Moreover, during the luteal phase, our progesterone peak (16.38 ± 5.88 ng/mL) was greater than that observed in Caucasian women with 10.2 ± 4 ng/mL (Rodriguez et al. 2001) or 5.2 ng/mL (Nakagawa et al. 2006). The absence of change in QT during the menstrual cycle in Caucasian women with low estradiol levels led some authors to suspect that at very high rates, estradiol could change cardiac repolarization. Their hypothesis was supported by Drici et al. (1996) who demonstrated a prolongation of the QT interval in ovariectomized rabbits exhibiting high estradiol concentration (302 ± 58 pg/mL). Estradiol was suspected to decrease the expression of potassium rectifier channels, therefore, slowing the repolarization phase of the cardiac action potential and prolonging the QT interval (Drici et al. 1996; Ebert et al. 1998).

This study was conducted in black African women, who have high level of estradiol, in agreement with the report of Marsh et al. (2011) and close level of estradiol than the experimental values reported by Drici et al. (1996). However, despite these high values of estradiol, we found no difference in QT interval during the menstrual cycle. For Kondo et al. (1989) and Saeki et al. (1997), subsequent increases in endogenous levels of FSH (Follicle Stimulating Hormone) and LH (Luteinizing Hormone) at the end of the follicular phase and increased level of progesterone during the luteal phase could inhibit the effects of estradiol on QT interval duration. Furthermore, Rodriguez et al. (2001), after administration of ibutilide, reported greater prolongation of QT interval during the first half of the menstrual cycle when estradiol levels were high and progesterone levels were low. We cannot exclude that oestrogens may predispose to a greater sensitivity to drug‐induced QT prolongation. However, this study was not designed to answer this question. Finally, Nakagawa et al. (2006), in 11 Holter ECG of women taken during their daily activities, reported significantly shorter QT interval in the luteal phase. Then, they suggested the role of sympathetic tone. Indeed, beta‐adrenergic stimulation modulates some ionic currents such as the Ica_L_^2+^, a slow‐activating component of the delayed rectifier potassium current and current Na/K pump current (Lindemann and Watanabe 1990; Nakagawa et al. 2005) that regulate the repolarization phase of the action potential of the cardiac cells.

It is recognized that the QT correction formulae give different values of corrected QT (Luo et al. 2004). Bazett's formula (1920) is known to give high corrected values compared to Fridericia's formula (Sagie et al. 1992; Charbit et al. 2006). The Fridericia's formula, giving better corrections than Bazett's formula, has been recommended by some authors (Funck‐Brentano and Jaillon 1993; Davey 2002). In the studies which did not show any influence of the menstrual cycle on the QT interval duration, the authors have used Bazett's formula (Burke et al. 1997; Rodriguez et al. 2001; Endres et al. 2004), Fridericia's formula (Rodriguez et al. 2001) and or specific regression parameters (Hulot et al. 2003). Among our subjects, the duration of the corrected QT interval was significantly higher with Bazett's formula than Fridericia's formula during the three phases of the menstrual cycle. Bazett formula gave QT values always significantly correlated to values of RR intervals, unlike the Fridericia formula. Values of QT corrected by these two formulae were correlated but were not interchangeable. Also, for studies on QT corrected value, the choice of Bazett's formula alone appears inadequate.

## Conclusion

At rest, this study showed that there was no significant variation in the duration of uncorrected QT and corrected QT intervals between menstrual, follicular, and luteal phases of the menstrual cycle among young black African woman. High estradiol level during follicular phase and high progesterone level in the luteal phase, in the young African woman did not influence the duration of uncorrected QT and QT corrected by the formulae of Bazett and Fridericia. Due to the absence of the interchangeability between Bazett's and Fridericia's formulae, the simultaneous use of at least two correction formulas of the QT interval duration is recommended in studies.

## Conflict of Interest

The authors have no conflicts of interest.
